# Do EU Member States Care About their Diasporas’ Access to Social Protection? A Comparison of Consular and Diaspora Policies across EU27

**DOI:** 10.1007/978-3-030-51245-3_1

**Published:** 2020-10-31

**Authors:** Jean-Michel Lafleur, Daniela Vintila

**Affiliations:** 1 FRS-FNRS & Centre for Ethnic and Migration Studies (CEDEM), University of Liege, Liege, Belgium; 2 Centre for Ethnic and Migration Studies (CEDEM), University of Liege, Liege, Belgium; 3 FRS-FNRS & Centre for Ethnic and Migration Studies (CEDEM), University of Liege, Liege, Belgium; 4 Centre for Ethnic and Migration Studies (CEDEM), University of Liege, Liege, Belgium

## Abstract

Despite the growing literature on sending states’ engagement with their populations abroad, little is known so far about their role in helping the diaspora deal with social risks. As argued in this chapter, this is mainly because past studies on sending states’ policies and institutions for the diaspora have failed to systematically focus on social protection, while also ignoring that regional integration dynamics often constrain domestic responses to the welfare needs of nationals residing abroad. This volume aims to fill this research gap by comparatively examining the type of diaspora infrastructure through which EU Member States address the vulnerabilities faced by populations abroad in five core areas of social protection: health care, pensions, family, unemployment, and economic hardship. Drawing on data from two original surveys with national experts, we operationalize the concepts of descriptive infrastructure for non-residents (i.e. the presence of diaspora-related institutions) and substantive infrastructure (i.e. policies that provide and facilitate access to welfare for nationals abroad) in order to propose a new typology of states’ engagement with their diaspora in the area of social protection.

## Introduction

Do sending states care about the well-being of their citizens residing abroad? In recent years, numerous studies have examined sending states’ policies and institutions targeting non-resident nationals. To underline the fact that such policy arrangements and initiatives generally concern individuals sharing some form of heritage with a homeland of which they may or may not hold nationality, they tend to refer to this population as diaspora (Adamson 2019). In documenting the growth in sending states’ activism and creativity in engaging with this population, scholars have identified several explanatory variables including increasing mobility, economic dependence on migration (especially remittances), democratization, the desire to gain political support from citizens abroad, or a shift to neo-liberal modes of government (Ragazzi 2014).

In this introductory chapter, we argue that existing attempts to classify states’ engagement with citizens abroad face four important limitations. First, past studies focused mainly on policy innovations developed by sending states to engage with citizens abroad in areas such as citizenship, education, business, culture or religion. This hinders the possibility of generalising existing classifications to other specific policy areas that are of key interest for the diaspora, such as the one of social protection. While recent work has acknowledged the existence of sending states’ policies aiming to respond to the social risks faced by non-resident citizens (Delano 2018), the role of welfare institutions in their design and implementation has not received sufficient scholarly attention. Second, existing studies do not engage sufficiently with the concept of consular assistance that, despite the limitations set by the 1963 Vienna Convention on Consular Relations,^1^ still varies greatly in its availability and content across states. Third, whether it draws on small or large-N studies, past research mainly focused on sending states from the Global South, therefore failing to notice developments in this area in the North and particularly among European Union (EU) Member States. Finally, the focus on the nation state overlooks the fact that sending states’ ability to respond to the needs of citizens abroad can be seriously constrained or triggered by regional integration dynamics (such as the EU), intergovernmental bodies (such as the International Organization for Migration) or complemented by policies adopted by sub-national public entities.

This volume focuses on EU Member States’ engagement with their diaspora in the field of social protection. To do so, we use the concept of diaspora infrastructure to identify how engaged sending states are in addressing the social risks faced by populations residing abroad in five key areas of social protection: health, employment, old age, family, and economic hardship. For each EU Member State, authors closely examine the core policies by which consular, social affairs-related ministries and ad-hoc diaspora institutions address risks in those areas. To highlight the variation in countries’ engagement with their diaspora in the field of welfare, this volume insists particularly on policies that go beyond the EU framework of social security coordination as established by Regulations No. 883/2004 and 987/2009.^2^ Overall, the objective of this introduction and the 27 country chapters^3^ included in this volume is to reconsider the meaning of sending states’ policies for nationals abroad and provide an alternative typology of their engagement by taking into account the array of policies and institutions through which they deal with social protection issues faced by their diaspora.

## Conceptual Framework: Bridging the Gap Between Consular Assistance, Diaspora Policies and the External Dimension of Social Security in the EU

### Whom to Protect? Diaspora and Citizenship in the EU

Looking at the success of the concept of diaspora in the study of the relation that migrants maintains with their homeland, some scholars have noted that this notion is regularly described as over-used and under-theorized (Anthias 1998). Following a period of heavy proliferation of the term, scholars such as Dufoix (2008) or Brubaker (2017) have stressed the confusion around the concept. Brubaker (2017) however, argued that it matters less to clearly identify what constitute a legitimate use of the concept than to acknowledge the existence of narrower and broader ways of using this notion. Such variations rely on the meaning given to its three core constitutive characteristics: dispersion, orientation towards the homeland, and relations with the host society. The country chapters included in this volume demonstrate that states define their diaspora very differently and this definition naturally influences the type of policies they adopt. For instance, the chapter on France shows how the French government has developed specific social programmes for nationals residing abroad in situation of need and/or unable to join destination countries’ social protection schemes. The extension of state-sponsored solidarity towards non-residents is therefore justified as a privilege associated to citizenship. On the contrary, several Central and Eastern European countries such as Hungary or Slovakia (see country chapters in this volume) also developed policies for individuals considered as part of their diaspora based on ethnic or cultural criteria. However, in the case of dual nationals or individuals who gave up their nationality while acquiring the citizenship of another country, the incentive for the homeland to engage in welfare may be more limited, as these individuals can access their residence countries’ social protection system. In this scenario, homeland authorities may consider cultural or return policies— more than social policies— as critical instruments to maintain or strengthen links with co-ethnics residing abroad.

**Fig. 1.1:**
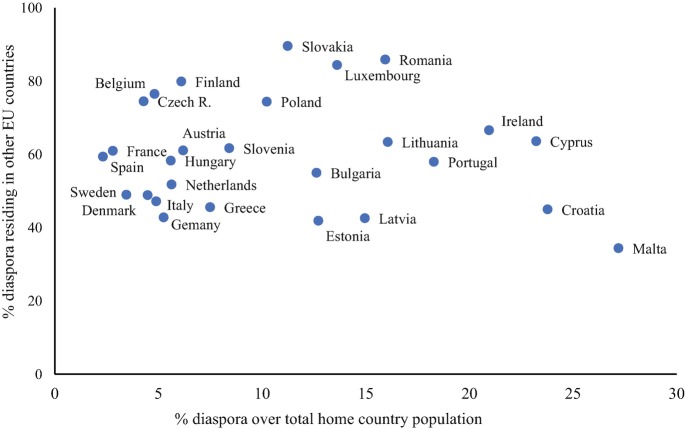
Diaspora populations of EU Member States: share of diaspora over total population and percentage of diaspora residing in the EU from the total diaspora population Source: Own elaboration based on OECD data. The data on diaspora stocks are from OECD (2015) “Connecting with emigrants: a global profile of diasporas 2015”, covering emigrant population (defined as foreign-born individuals by country of birth and their children born in destination countries) aged 15+ across 84 selected destinations (33 OECD countries and 51 non-OECD states). For Malta and Cyprus, diaspora stocks are from the DIOC-E 2010/2011 Labour Force Status dataset, covering emigrant population aged 15+ across 87 destinations (35 OECD countries and 52 non-OECD states). The data on total population are from the OECD Historical Data file (population 15+, reference year 2010, https://stats.oecd.org/Index.aspx?DataSetCode=POP_PROJ#, accessed 16 March 2020). The data on diaspora populations residing in EU countries are from the DIOC-E 2010/2011 Labour Force Status (thus including the UK as an EU destination country). However, this source does not always provide information on the diaspora population of each Member State residing in other EU countries. This information is missing for the following groups: Austrian and Slovakian diaspora residing in Bulgaria, Lithuania, and Romania; Belgian and Irish diaspora in Austria, Bulgaria, Germany, and Lithuania; Croatian diaspora in Cyprus, Lithuania, Malta, and Romania; Czech diaspora in Malta and Romania; Cypriot diaspora in Austria, Germany, Lithuania, and Romania; Danish and Latvian diaspora in Austria, Bulgaria, Germany, and Romania; Estonian diaspora in Austria, Germany, Malta, and Romania; Finnish and Swedish diaspora in Austria, Bulgaria, Germany, Lithuania, and Romania; French and Spanish diaspora in Austria and Lithuania; Greek diaspora in Austria and Malta; Italian diaspora in Bulgaria and Lithuania; Lithuanian diaspora in Austria, Bulgaria, Romania, and Malta; Luxembourgish diaspora in Austria, Bulgaria, Cyprus, Germany, Latvia, Lithuania, Romania, and Malta; Maltese diaspora in Austria, Bulgaria, Estonia, Germany, Lithuania, and Romania; Dutch diaspora in Austria, Bulgaria, and Lithuania; Portuguese diaspora in Austria, Bulgaria, Lithuania, and Romania; Slovenian diaspora in Bulgaria, Germany, Lithuania, and Malta

The perimeter of EU Member States’ diaspora engagement strategies is further blurred by three additional elements. First, because of the different historical, political, and socio-economic contexts in which emigration from EU countries has taken place, this phenomenon is not equally salient across all Member States. Variations in the demographic weight of the diaspora – often derived from the different timing of migration outflows- still exist, thus representing an important contextual element for examining states’ engagement with this population. As shown in Fig. 1.1, the relative size of the diaspora over the total population of each EU Member State varies greatly, from less than 3% in Spain or France to 15% or more for Latvia, Romania, Lithuania, Ireland, Cyprus, Croatia or Malta. Of course, timing of emigration is a particularly relevant aspect here. Countries with longer history of emigration (e.g. Italy, Ireland, Spain, Greece, Finland) naturally have had more time to respond to these significant outflows by implementing policies for citizens abroad compared to newer emigration countries (especially Member States from Central and Eastern Europe).

Second, EU Member States have to deal with different categories of nationals residing abroad who potentially have different social protection needs, depending on their countries of residence. On the one hand, there are those residing in other EU Member States. This first group benefits from the EU citizenship status and associated rights, including the right to free movement and residence in the EU, as well as the EU legislation on equal treatment and social security coordination. As shown in Fig. 1.1, more than 75% of the diaspora population of Belgium, Finland, Luxembourg, Romania, and Slovakia are intra-EU migrants. These countries may thus have fewer incentives to develop diaspora and consular policies in the area of welfare since the vast majority of their non-resident nationals are, in any case, covered by the EU legislation. Yet, as noted by Ragazzi (2014), existing diaspora studies tend to neglect regional integration as a form of state engagement with citizens abroad. This entails that our current understanding of who is a “protective” state for its diaspora and who is not does not take the reality of EU integration into consideration.

Figure 1.1 also points towards a second cluster of EU Member States (including Malta, Estonia, Latvia, Germany, Croatia, Greece, Italy, Denmark, and Sweden) for which more than a half of their diaspora resides in non-EU destinations. These states’ engagement with non-resident nationals in the area of welfare is often limited to basic consular services (themselves regulated by the 1963 Vienna Convention), a right to be helped by consular authorities of other EU countries (deriving from the Directive on consular protection for EU citizens living or travelling outside the EU^4^) and social security agreements signed with third countries. Less frequently, EU citizens residing in non-EU countries can benefit from ad-hoc social protection policies designed for the diaspora and/or maintain some access to homeland welfare benefits (see the discussion on substantive infrastructure below).

Third, beyond the distinction between EU and non-EU destination countries, diaspora populations tend to concentrate in a handful of countries of residence. Table 1.1 displays the top five destination countries of each EU Member State’s diaspora. Interestingly, more than a half of the Irish, Finnish or Slovak diaspora is concentrated in a single country. Less surprisingly, some large Western democracies such as the United States of America (USA) or Canada have become important destinations for the diaspora population of several EU countries, whereas Germany and the United Kingdom (UK) rank as top host countries for more than 60% of the non-resident population of other EU Member States. Concentration of the diaspora, we argue, is an important element that could shape states’ policies towards their nationals abroad. More specifically, concentration and mobilization of the diaspora in one host country in particular may push homeland authorities to adopt tailored-made policies that apply only to citizens residing in that country (as opposed to developing policies for all non-resident nationals, regardless of their destination countries). Chapters included in this volume therefore take the precaution of specifying the geographical scope of policies when they are restricted to certain destination countries.

**Table 1.1:**
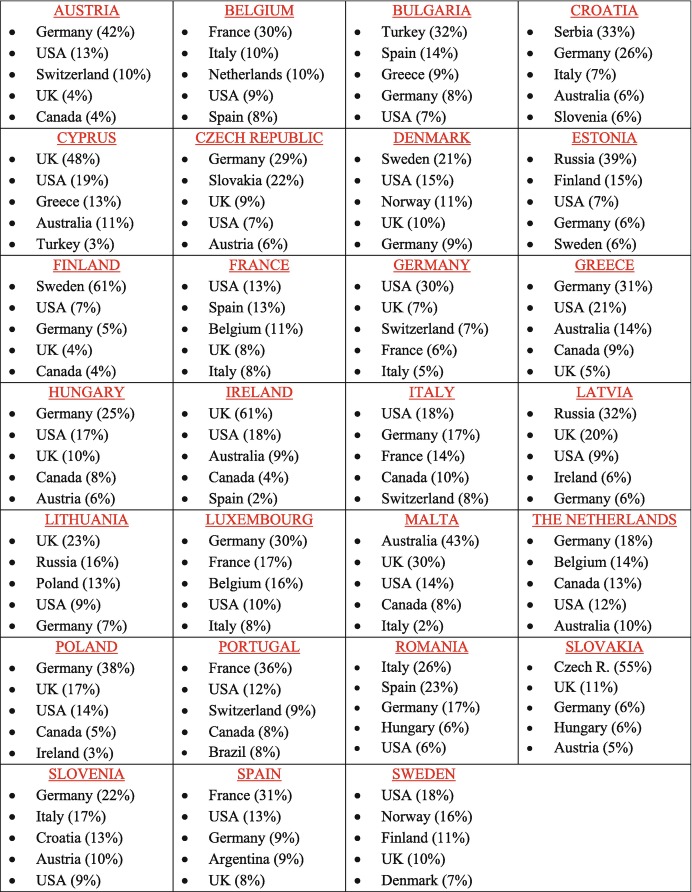
Main destination countries of the diaspora populations of EU Member States, by share of diaspora in each host country from the total diaspora of each Member State

Source: Own elaboration based on OECD (2015) “Connecting with emigrants: a global profile of diasporas 2015”. The data refers to emigrant populations (defined as foreign-born individuals by country of birth and their children born in destination countries) aged 15+ across 84 selected destinations (33 OECD countries and 51 non-OECD states). For Malta and Cyprus, diaspora stocks are from the DIOC-E 2010/2011 Labour Force Status dataset, covering emigrant populations aged 15+ across 87 destinations (35 OECD countries and 52 non-OECD states)

### How to Protect? Consular and Diaspora Policies for EU Citizens

In the previous section we have called for a broadening of the definition of states’ engagement with nationals abroad, to take into account different types of sending states’ social protection interventions. In prior attempts to measure states’ commitment with populations abroad, scholars have coined new concepts that move partially or fully away from an exclusive focus on diaspora policies. Unterreiner and Weinar (2017: 11), for instance, distinguish immigration policies from emigration policies, which they define as “*all policies that regulate (either facilitate or limit) outward migration, mobility across countries and possible return*”. Although this categorization is conceptually attractive, it however neglects that certain policies (such as bilateral social security agreements) are often both emigration policies through which sending states facilitate physical relocation (e.g. by allowing pension contributions in home countries to be recognized in host countries) and immigration policies through which receiving states aim to facilitate integration by limiting individuals’ exposure to social risks. Clear-cut distinctions are thus not obvious.

In line with the literature that focuses on intentionality, Unterreiner and Weinar (2017) further distinguish diaspora policies as “*policies that engage emigrants and members of diaspora communities (both organised groups and individuals) with the countries of origin, building a sense of belonging and strengthening ties*”. Their definition of diaspora policies is therefore close to what Pedroza et al. (2016: 14) understand as emigrant policies, that is “*policies that states develop specifically to establish a new relationship towards, or keep links with, their emigrants*”. For Pedroza and colleagues, emigrant policies therefore exclude the hard-to-distinguish host states’ immigration policies, home states’ policies enabling departure and, most importantly, most consular tasks as defined by the Vienna Convention on Consular Relations.

Surprisingly, with the exception of the work of Delano (2013, 2018), the role of consulates in assisting emigrants to deal with risks abroad has not received significant scholarly attention. So far, the literature has assumed that, while important cross-country variations in the presence of consulates exist, services are broadly similar and limited to: strengthening commercial, economic, cultural, and scientific relations between home and host countries; issuing passports and travel documents; serving as a notary and civil registry; and assisting detained nationals abroad (Aceves 1998). These missions derive from article 5(e) of the Vienna Convention that vaguely defines consular functions as “*helping and assisting nationals, both individuals and bodies corporate, of the sending State*”. For Okano-Heijmans (2010), the concept of ‘consular affairs’ is commonly used to refer to assistance to non-resident citizens in distress, but states tend to leave these concepts open to interpretation which, *de facto*, leaves significant discretionary power to consulates in dealing with citizens abroad. The lack of conceptual clarity in the definition of consular services and the fact that the delivery of certain services is sometimes left at the discretion of authorities renders the comparison between EU Member States difficult. Accordingly, when examining consular policies (along with other diaspora policies), this volume focuses primarily on policies based on norms adopted by legislative and/or executive-level homeland authorities; and discretionary measures and administrative practices are only mentioned for illustrative purposes.

In the case of EU countries, significant attention has also been paid to consular functions exercised by any EU Member State for EU citizens living in third countries in which their state of nationality is not represented. Council Directive 2015/637 stipulates that consular assistance is limited to cases of: death, serious accidents or serious illness, arrest or detention, being a victim of crime, relief and repatriation in case of emergency, and the need for emergency travel documents (see Faro and Moraru (2010) for an in-depth discussion of consular practices of EU countries). However, the emphasis on this specific policy - presented as a response to the needs of EU citizens residing in third countries- is limitative in two ways. First, it overlooks the fact that consulates may play a critical role in their nationals’ access to social protection even within the EU. As discussed by Palop-García (this volume) or Nica and Moraru (this volume), the presence of Romanian and Spanish social affairs *attachés* in different consulates throughout the EU is a testimony of the relevance of such consular actors whose presence and activities aim to reduce practical inequalities in access to welfare. Second, consular services of many Member States are moving away from a model based on physical presence in destination countries to a more diverse offer that also includes e-services and mobile consular services (i.e. temporary detachment of consular personnel) in cities where no consulate is present. Overall, this brief discussion on consular services in the EU highlights the necessity for our 27 country chapters to provide a deeper analysis of the physical availability (and variations in content) of consular services for EU citizens in situation of international mobility, whether they live inside or outside the EU.

### What Kind of Protection? Deterritorialized Social Security as Protection Policy for EU Citizens Residing Abroad

Facing difficulties in accessing benefits in the host country and loosing entitlements gained previously in their home country are frequent issues met by international migrants. State cooperation in the area of welfare can address these problems, although this cooperation is often hindered by varying conditions of access to benefits across states and their different funding schemes. Even within the EU, specific benefits can be contribution-based in one Member State and simply not exist or be tax-financed with severe means testing in another (see Lafleur and Vintila 2020a in this series). When it comes to accessing public healthcare or contributory pensions, for instance, mobile EU citizens benefit from the most advanced regime of state cooperation to deal with the social risks of individuals in situation of international mobility (Holzmann et al. 2005; Avato et al. 2010). This privileged position when compared to other international migrants is further reinforced by the legal framework on non-discrimination, equal treatment, and the right to reside applicable across the EU. In other words, in the process of encouraging labour mobility to achieve the Single Market (Maas 2013), EU Member States have contributed to the deterritorialization of their social protection systems. As a result, residence outside the territory of a specific welfare state stopped being an obstacle to maintain some form of access to social benefits from that state. Portability and exportability of welfare entitlements are thus key features of this deterritorialization process.

Portability is one’s ability the preserve, maintain and transfer acquired social security rights in areas such as pensions or healthcare, independently of one’s nationality or residence country (Holzmann et al. 2005). Welfare authorities of migrants’ sending and receiving states typically tend to agree on portability of pension entitlements to ensure that individuals with a history of international mobility who have paid contributions in different countries are not deprived from accessing pensions. For EU citizens overseas who do not benefit from the EU legal framework on pensions,^5^ a number of international treaties and conventions from institutions such as the International Labour Organisation or the United Nations^6^ are designed to set minimum standards and encourage— with little binding force— good global practices. Nonetheless, because of the lack of coordination in the external dimension of EU social security,^7^ portability rights of EU citizens living outside the EU still depend on Member States’ ability to enter social security agreements with third countries. In this volume, country chapters explicitly discuss such agreements and show that almost all Member States have signed bilateral or multilateral social security agreements with the third countries that represent the main destinations for their diaspora.

Exportability refers to individuals’ ability to receive a particular benefit to which they are entitled while residing outside of the territory of the welfare state that pays for it. Here again, pensions are, by far, the most commonly accepted form of exportable benefit (Holzmann et al. 2005; Vintila and Lafleur 2020). Country chapters in this volume also show that bilateral agreements between EU Member States and third countries tend to include pension exportability. However, only contributory pensions tend to be exportable, as non-contributory pensions are frequently reserved for residents. Similarly, some Member States may reduce the amount of pensions when beneficiaries reside in specific third countries (Pennings 2020).

Regulation 883/2004 on social security coordination provides further illustrations of the fact that mobile EU citizens residing in other Member States have access to a more favourable exportability regime when compared to EU nationals residing in third countries. For instance, the Regulation allows EU citizens moving to another Member State for the purposes of finding a job to export unemployment benefits for three months (up to a maximum of six months).^8^ It also explicitly envisages the exportability of family benefits when the country where the parent works and the country where the child resides are not the same.^9^ For EU citizens moving outside the EU, on the contrary, the assumption is that their access to family benefits will be determined by the host country’ regulations and, when applicable, bilateral/multilateral agreements. Additionally, the European Health Insurance Card (EHIC) also allows EU nationals to access state-provided medical healthcare during temporary stays in other EU Member States, Iceland, Liechtenstein, Norway and Switzerland, under the same conditions and at the same costs as individuals insured in those countries.^10^ Beyond these examples, only few benefits are exportable; and in general, non-contributory benefits are typically designed to respond to the needs of residents (Vintila and Lafleur 2020). Yet, in the next section, we highlight the fact that several Member States have adopted specific responses to the social protection needs of their diaspora.

## Diaspora Infrastructure

In this section, we use the concept of diaspora infrastructure to compare EU sending states’ diaspora institutions and policies that address the social protection needs of their non-resident nationals. As discussed, existing conceptualizations of sending states’ policies do not capture adequately the specificities of EU Member States, while also overlooking origin countries’ policies in the area of welfare. Past studies usually distinguished between two types of diaspora institutions (Agunias and Newland 2012; Gamlen 2019). First, there are government-led bodies such as ministries, sub-ministries or agencies functioning as administrations which respond to the specific needs of populations abroad or maintain a connection (of economic, cultural or political nature) with non-residents. Second, other bodies function as consultative or representative institutions of the diaspora and often include members from the diaspora via election or appointment. Their function is generally to defend diaspora’s interests in the home country’s policy-making process. Sending states’ institutions that enable citizens abroad to access host or home countries’ welfare benefits have therefore often been overlooked in the literature.

The concept of infrastructure has experienced a growing use in migration studies with the literature on “arrival infrastructure” studying the interaction between the local environment and immigrant integration (Meeus et al. 2019). Anthropologists such as Kleinman (2014) also refer to infrastructure to describe both the physical environment and the web of social interactions that allow precarious migrants to get by. With the concept of diaspora infrastructure, we aim to highlight the fact that sending states’ engagement with nationals abroad in the area of welfare consists of both institutions (consulates, ministries or sub-ministries in charge of emigration issues) and policies (rights and support services) aiming to protect the diaspora against vulnerability or social risks.

Confronted with the diversity of home country institutions and policies relevant for citizens abroad, we have chosen to articulate the notion of diaspora infrastructure based on two different (but sometimes interconnected) conceptual dimensions. Inspired by the literature on political representation of minorities (see Pitkin 1967; Phillips 1995; Powell 2004; Bird et al. 2011, among others^11^), we distinguish between descriptive and substantive state infrastructure for nationals abroad. Considering the well-documented trend among sending states to engage only symbolically with their diaspora by creating institutions that perform limited tasks or by adopting policies with limited impact on diaspora’s welfare (Gamlen 2019), the distinction between descriptive and substantive infrastructure is particularly appealing to qualitatively assess sending states’ engagement. In our view, descriptive infrastructure captures the extent to which sending states create an institutional setting that specifically targets the diaspora in its scope and aims. This concept captures the “presence” of homeland institutions that explicitly acknowledge the diaspora as main reason for their existence, while formally being granted the mission to act in its interests (including welfare-related interests). As discussed below, descriptive infrastructure may include a sending country’s consular network, but also ministries, sub-ministries, agencies or representative bodies that perform a public mission in the interest of the diaspora.

Substantive infrastructure, on the other hand, refers to the existence of policies in the area of social protection by which sending states provide rights and services that address diaspora’s social risks. As we show below, an extensive substantive infrastructure can be measured not only by the diaspora’s ability to benefit from some level of coverage from the home country’s welfare state, but also by the capacity of sending states’ authorities to provide practical support to nationals abroad who are in need. Of course, having an extensive descriptive infrastructure does not necessarily mean that states also adopt extensive policies through which they actively respond to diaspora’s social protection needs, as specific diaspora institutions may be created only symbolically while still veiling a rather superficial sending states’ responsiveness to the concerns of nationals abroad. Alternatively, states may still be able to ensure a comprehensive substantive infrastructure for non-resident populations even in absence of a widespread institutional network formally working in the interest of the diaspora. Yet, the mere existence of an extensive public structure of institutions can still carry an important symbolic weight, as it may be considered as a formalised recognition of diaspora’s importance for the homeland. An extensive descriptive infrastructure is thus expected to be correlated with an extensive substantive infrastructure, although it is not a sufficient, nor a necessary condition, for the latter.

From an empirical viewpoint, our assessment of descriptive and substantive diaspora infrastructure relies on two large-N datasets designed in the framework of the ERC-funded project “Migration and Transnational Social Protection in Post (Crisis) Europe” (MiTSoPro).^12^ The diaspora policy dataset was created by collecting a large amount of data on national policies,^13^ using a standardized questionnaire filled by experts on consular and diaspora policies across 40 countries (including the EU27 Member States analysed here). In our description of substantive infrastructure, we also use some data on welfare entitlements of citizens abroad from a second MiTSoPro dataset on access to social protection, drawing on a second survey on national social protection policies with social policy experts across the same 40 countries (see Vintila and Lafleur 2020 for further details).

### Descriptive Infrastructure: Consular, Governmental, and Interest-Representation Institutions

As previously mentioned, we operationalise sending states’ descriptive infrastructure as the institutional framework that comprises home countries’ public institutions at the national level which meet both conditions of having a mandate to engage primarily with the diaspora and being active in the adoption or implementation of social protection policies that benefit this population. Institutions that form the descriptive infrastructure can have either direct relations with the diaspora (e.g. when an institution provides the diaspora with a specific service/benefit) or indirect ones (i.e. when it only participates in the design of diaspora policies). Similarly, some of these institutions can be solely present physically in the home country, while others can operate in (all or selected) countries of residence. Regardless of the intensity of their interactions with the diaspora or the main location of their activities, all the institutions that compose a country’s descriptive infrastructure however share the characteristic of performing a public mission that contributes to addressing diaspora’s social protection needs.

The use of this specific definition of descriptive infrastructure has two important implications for assessing how protective states are towards their non-resident populations. First, by focusing on public institutions with a legal mandate to govern or administrate states’ relations with the diaspora, the limited number of EU Member States, such as Ireland, that usually fund non-state actors (e.g. migrant associations) to perform missions of assistance to the diaspora may appear as less engaged. Similarly, because we focus on national institutions, the limited number of sub-national institutions that exist in some EU countries are also excluded from our measurement of descriptive infrastructure. However, when relevant, both regional actors and state-funded non-state actors are discussed in the country chapters for illustrative purposes.

**Fig. 1.2:**
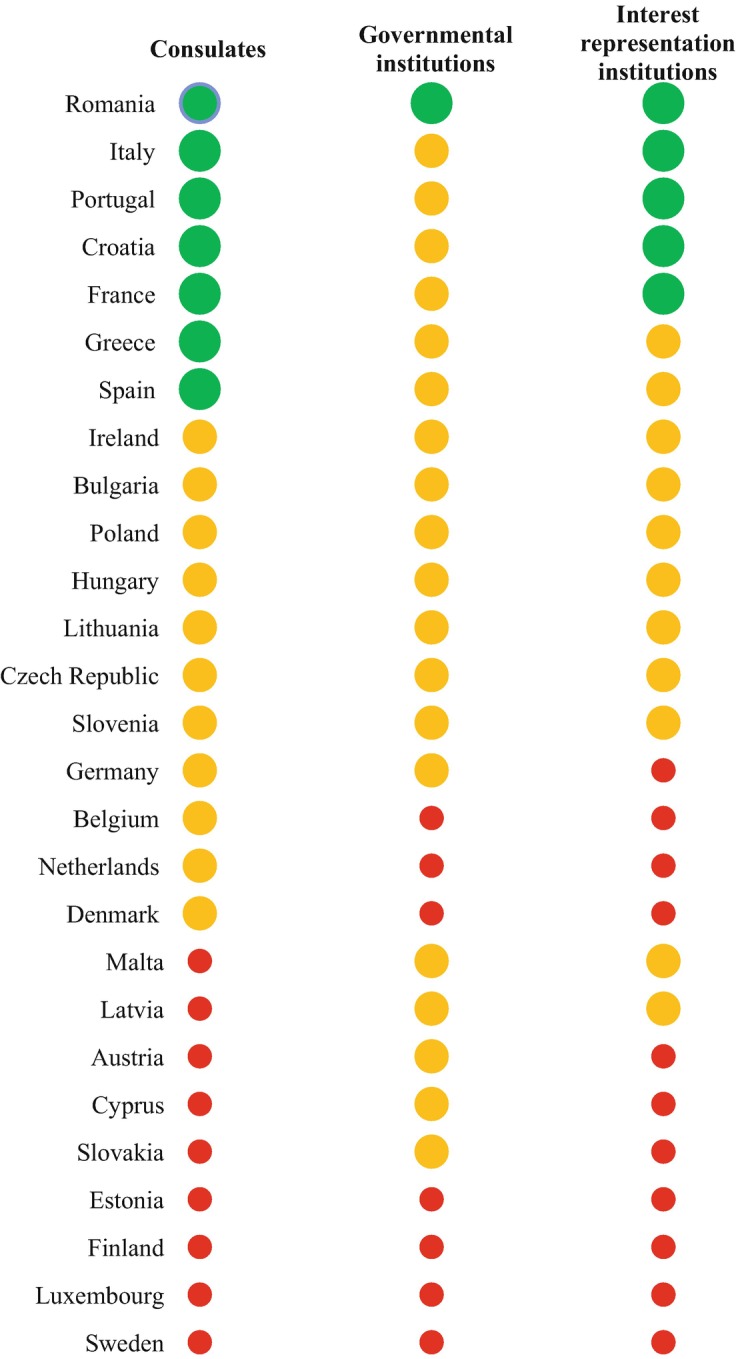
Descriptive infrastructure: presence of diaspora-related public institutions of EU Member States Source: Own elaboration based on MiTSoPro data. Consular presence is considered as extensive (green) when a country has 20+ consulates in top five destination countries; moderate (yellow) when the number of consulates is between 10 and 19; and limited (red) when the number of consulates is lower than 10. Regarding the network of governmental institutions for the diaspora, we consider it as extensive (green) for countries with at least a ministry for the diaspora; moderate (yellow) for countries with only sub-ministerial institutions; and absent (red) for countries that have neither type of institutions. Interest-representation institutions are measured as extensive (green) when a country has at least reserved seats in the national Parliament for diaspora representatives; moderate (yellow) when it has only consultative institutions for nationals abroad; and none (red) when neither of these interest-representation institutions exist

Drawing on this definition and the information provided by the country chapters in this volume, Fig. 1.2 shows a comparative overview of the descriptive infrastructure that EU countries put forward for their diaspora. The Figure captures three types of institutions that are analysed below: a) consulates; b) governmental institutions (covering ministry and sub-ministry level institutions for non-residents) and; c) interest-representation institutions (either at the legislative or consultative level). As observed, there is substantial variation across EU countries in the repertoire of institutions they create to engage with the diaspora. Some Member States (especially Romania, Italy, Portugal, Croatia, France, Greece, and Spain) show a higher variety of institutions dealing with non-residents when compared to other countries (particularly Estonia, Finland, Luxembourg or Sweden), which return a very limited descriptive infrastructure for nationals abroad.

#### Consular Network

As noted previously, consulates perform different missions for citizens abroad that are relevant for their access to welfare. These missions range from the delivery of indispensable documents to access certain benefits (e.g. life certificate to continue receiving a home country pension while abroad), direct provision of benefits (e.g. consular financial assistance in case of exceptional hardship), information provision on home and host countries’ welfare systems (e.g. on their website, via brochures or information sessions) and, more exceptionally, assistance to access benefits (see below). The country chapters included in this volume provide details that point towards an important variation between EU Member States in the type of services they offer. Some also discuss how certain EU countries have engaged in the deterritorialization of their consular services by offering mobile consular services (i.e. physical movement of consular staff to locations where no consulate is present) or by allowing some consular services to be delivered electronically without the need for citizens to move.

**Fig. 1.3:**
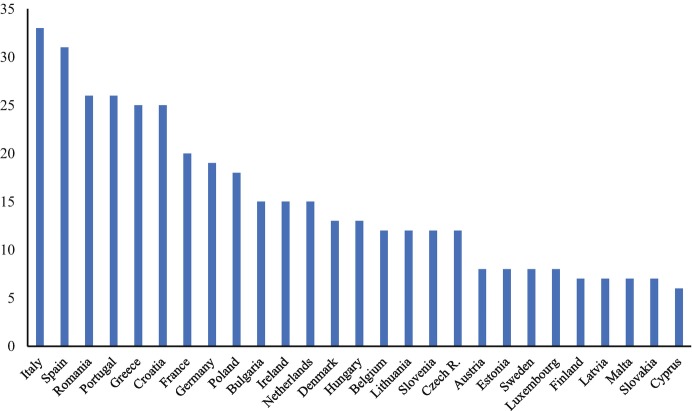
Consular presence of EU Member States in top five destination countries (total number) Source: Own elaboration based on MiTSoPro data

Figure 1.3 identifies the “physical presence” of consulates in destination countries, defined as the total number of consulates that each EU Member State has in the top five residence countries of their diaspora. Although some honorary consulates also offer limited administrative services to citizens abroad, we excluded them from the analysis, thus focusing exclusively on consulates offering the widest range of consular services in each Member State’s consular law.^14^ This approach of focusing on the five largest destination countries of EU Member States’ diaspora populations is in line with our concept of “descriptive infrastructure” whose core idea is that the presence of homeland institutions should be reflective of the presence of citizens abroad. Of course, this approach also faces certain limitations. For instance, there may be reasons to open a consulate— such as the desire to increase trade, cultural or political relations with a particular country— that are not necessarily related to the presence of the diaspora. Also, when a large share of the diaspora in a particular destination country already holds that country’s nationality or shows high levels of socio-economic integration, the incentive of sending states to open/maintain consulates in that specific destination country may be weaker. Lastly, the geographic size of destination countries and diaspora’s concentration in the territory of those receiving states can further influence the presence of home country consulates.

Bearing in mind these limitations, Fig. 1.3 (and the part on consulates in Fig. 1.2) allow us to distinguish three clusters of EU Member States according to their consular presence. First, a group of seven Member States have at least 20 consulates in total in the top-five destination countries of their diaspora and can therefore be considered as returning an extensive consular presence. This group includes five countries from South and South East Europe with a long tradition of large scale emigration (Spain, Italy, Portugal, Greece, and Croatia), Romania (which started to experience substantial migration outflows especially since the 2000s), and one large former colonial power which has one of the most sizeable diaspora populations in absolute terms (France). A second cluster includes 11 North Western and Central and Eastern European countries that return a moderate consular network (between 10 and 19 consulates in top destination countries). The third cluster comprises nine Member States with more limited consular presence (less than 10 consulates in top destination countries). This group concentrates smaller EU countries (less than eight million inhabitants).

**Fig. 1.4:**
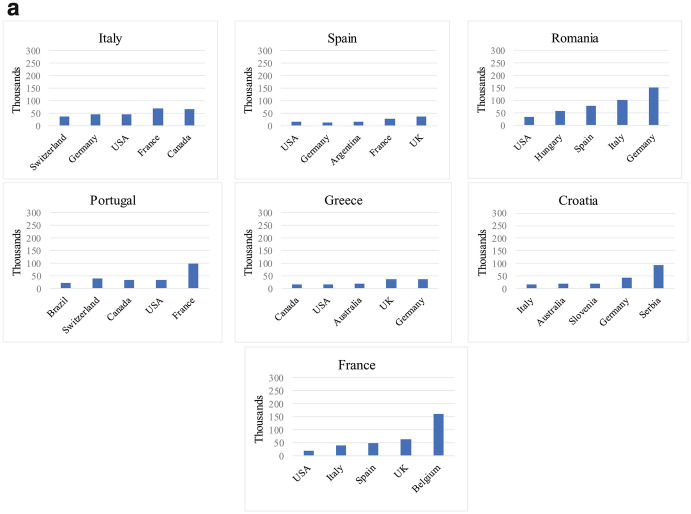

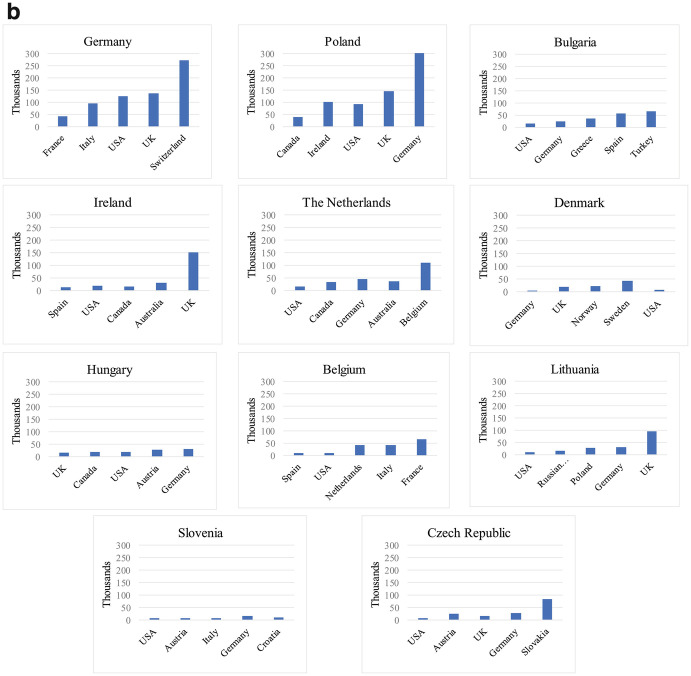

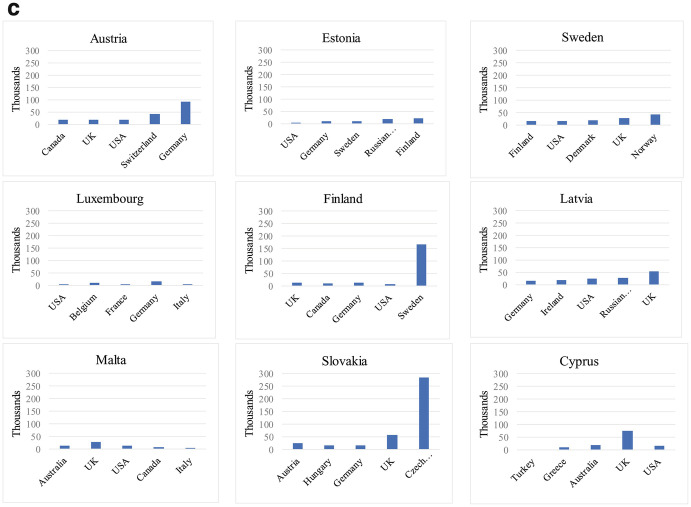
Ratio emigrants/consulates in top destination countries of EU Member States. (**a**) Member States with extensive consular network (20+ consulates in top destination countries). (**b**) Member States with moderate consular network (10–19 consulates in top destination countries). (**c**) Member States with limited consular network (less than 10 consulates in top destination countries) Source: Own elaboration based on MiTSoPro data. There is no consular representation of Cyprus in Turkey, hence this case appears with value “0”

Overall, while this classification gives us an indication of sending states’ willingness to be physically present where their diaspora concentrates, it does not tell us whether such presence is adequate considering the size of the diaspora in those countries. In Fig. 1.4, we propose an estimation of the adequacy of such consular presence by highlighting how many potential individuals the consular network of each EU Member State has to serve in the top five destination countries. For clarity purposes, the data is presented according to our typology of consular presence (extensive, moderate, limited, as explained above).

Two important patterns emerge. First, among the states with moderate or extensive consular networks, a group of four Member States (Romania, France, Germany, and Poland) have to serve potentially much more citizens per consulate than other countries in these clusters, this questioning their ability to face a particularly high demand of services. Second, among states returning a limited consular presence, we unsurprisingly find a majority of countries with limited diaspora presence in top five destinations, which somewhat justifies the rather small number of consulates they set up. Yet, we also find two Member States (Slovakia and Finland) whose nationals abroad concentrate mostly in one destination country, hence the demand of consular services in these specific states is much higher.

#### Governmental Institutions

The second category of institutions that are part of EU Member States’ descriptive infrastructure are governmental institutions for the diaspora. In line with the definition of Agunias and Newland (2012), these are homeland public institutions at the ministerial and sub-ministerial level whose legal mandate primarily consists in engaging with the diaspora and which design or implement policies aiming to respond to the perceived social protection needs of nationals abroad. To distinguish between ministry and sub-ministry level institutions, we rely on their criteria of “hierarchical independence” according to which only ministry-level institutions have stable financial means and can manage the diaspora portfolio in all its dimensions (Agunias and Newland 2012). Sub-ministry level institutions, in turn, are executive-level agencies or departments hierarchically dependent on ministries (typically, the Ministry of Foreign Affairs or the Ministry of Labour), but whose missions go beyond basic consular services set by the Vienna Convention.^15^ However, differences in the level of autonomy enjoyed by these institutions are not always reflected in their names. State secretaries, for instance, are autonomous from ministries in some countries, while being directly associated to or dependent on certain ministries in others. Hence, institutions with similar names sometimes belong to different categories of governmental institutions.

In Fig. 1.2, we considered Member States that have at least a ministry for the diaspora (which means that they can also have sub-ministerial institutions in addition to the ministry) as returning a strong network of governmental institutions. This choice is also justified by the fact that ministry-level institutions are undoubtedly an indication of the greater visibility that some EU countries wish to grant to the diaspora population. Following this approach, states that have only sub-ministry level institutions are considered as having a moderate offer, while those who have neither type as having no network of governmental institutions for nationals abroad.

Our comparative analysis reveals that, at the time of data collection (2019), Romania- which also represent one of the EU countries with the fastest growing emigrant population in recent years- was the only Member State with a ministerial body in charge of engaging with the diaspora. As explained by Nica and Moraru (this volume), the Ministry for Romanians Abroad was recently institutionalised (ten years after the country joined the EU), thus further extending the institutional network that the Romanian government has started to design for its diaspora even before the large emigration wave during mid-late 2000s. However, as noted in different country chapters, such ministries for the diaspora often tend to appear and disappear as new governments take power. This is the case of Italy and France, which had such ministry-level institutions in the past, but no longer do.

Although most Member States have not specifically created ministries aiming to address the needs of nationals abroad, the majority of them do have sub-ministerial institutions to represent diaspora’s interests. Such institutions are present across 19 EU Member States (Fig. 1.2), including countries with a long-standing emigration history such as Greece, Ireland, Italy or Spain, but also more recent emigration countries such as Poland or Bulgaria. These sub-ministerial institutions however enjoy varying levels of autonomy. As explained in the country chapters, some Member States have departments tasked with engaging with the diaspora, which are located within the Ministry of Foreign Affairs (e.g. Italy’s Directorate General for Italian Citizens Abroad and Migration Policies) and, occasionally, the Social Affairs Ministry (e.g. Spain). Such institutions usually benefit from less autonomy than ad-hoc agencies set up in a number of Member States. Lastly, only three states have sub-ministerial institutions in the form of political positions that grant their holders larger room for manoeuvre to design policies, while being hierarchically dependent on another ministry (see the Special Envoy for Expatriates of the Czech Republic, Ireland’s Ministry of State for the Diaspora and Latvia’s Ambassador for the Diaspora). Moreover, our findings also show that seven EU countries (Belgium, Denmark, Estonia, Finland, Luxembourg, the Netherlands, and Sweden) still consider that their bureaucratic dealings with the diaspora should be limited to basic consular services. Consequently, these countries have not designed ministerial or sub-ministerial institutions for their nationals abroad.

#### Interest-Representation Institutions

The third type of homeland institutions considered for our operationalisation of descriptive infrastructure are interest-representation institutions, i.e. home country public institutions with a legal mandate to voice diaspora concerns in the home and/or host country. Many chapters show how frequent it is for EU Member States to have institutions that officially allow representatives of the diaspora to communicate (in a non-binding way) their concerns in the homeland via assemblies, councils or forums. Yet, a handful of Member States also have interest-representation institutions organized at the destination country level, such as the Committees of Italians Abroad organized at the consular level to act as a link between the diaspora and consular authorities. By definition, interest-representation institutions are expected to cover a wide range of issues relevant for the diaspora (e.g. passport delivery, dual citizenship, access to culture, etc.), but they are also likely to include more niche welfare-related interests into the domestic political agenda of the homeland, as long as this is a relevant issue of concern for nationals abroad.

We distinguish between two types of interest-representation institutions. First, legislative-level institutions represent diaspora’s interests in the national Parliament (in either or both chambers, when applicable) through members of the Parliament (MPs) elected by voters residing abroad. In Fig. 1.2, we considered that EU Member States offering such legislative representation for the diaspora put forward an extensive infrastructure. As observed, five Member States currently allow their non-resident citizens to elect their own MPs (Croatia, France, Italy, Portugal, and Romania). This presence of elected MPs for the diaspora is an indication of the electoral visibility that states give to their nationals abroad, but the limited number of seats available for external constituencies also reveals the limited capacity that these constituencies actually have to influence the legislative process (see also Vintila and Soare 2018). Second, interest representation can also take the form of specific representative institutions whose role of defending diaspora’s interests is officially acknowledged in public policies adopted by homeland authorities. When compared to parliamentary seats for the diaspora, these representative bodies have far less visibility in homeland politics and policies, although they usually enable a dialogue between diaspora representatives and a multiplicity of homeland actors. For this reason, EU Member States that only have this type of bodies for their nationals abroad are considered to return a moderate type of interest-representation institutions in Fig. 1.2. The members of such bodies are either appointed by homeland authorities or elected by citizens abroad. While they are homeland public institutions, their mission of interest representation may be oriented towards the homeland and/or the countries of residence. Our results indicate that this type of representative bodies are present across 16 EU Member States in total;^16^ in 11 of them (see the cases marked in yellow in Fig. 1.2), such bodies constitute the only interest-representation institutions that states make available for non-residents. Our findings also show that, overall, 11 EU Member States do not count with any type of interest-representation institutions for their diaspora. This cluster (marked in red in Fig. 1.2) includes Austria, Belgium, Cyprus, Denmark, Estonia, Finland, Germany, Luxembourg, the Netherlands, Slovakia, and Sweden.

In addition to the consular, governmental and interest-representation institutions already captured under our umbrella concept of descriptive infrastructure, several chapters also mention other institutions that are still relevant for the diaspora populations of EU Member States. However, they have not been included in our definition of descriptive infrastructure as they fail to meet the double condition of having a primary mandate to engage with nationals abroad and participate in the design/implementation of policies aiming to respond to diaspora’s social protection needs. Among these institutions, some have prerogatives in the area of welfare, such as the presence of representatives of the Spanish Ministry for Social Affairs in specific consulates abroad. Others- quite common across all EU countries, except for Belgium, Malta, and Slovenia- are cultural institutions aiming to provide services abroad related to cultural, educational, linguistic or religious affairs of the home country (language courses, school networks supported with homeland’s funds, or general promotion of cultural activities abroad). Finally, several chapters also discuss the relevance of homeland parties operating abroad with the aim to defend diaspora’s interests in origin countries.

### Substantive Infrastructure: Sending States as Providers and Facilitators of Social Protection

In this section, we question the assumption that the existence of diaspora institutions is a sufficient condition to determine states’ engagement with nationals abroad in the area of social protection. We argue that descriptive infrastructure offers only a limited picture of how protective states are of the diaspora; and that a comprehensive assessment of their engagement with non-residents should also consider the content of homeland public policies that enable nationals abroad to deal with social risks, regardless of the characteristics of the institutions implementing such policies. We define the later as substantive infrastructure. We operationalise this concept via two dimensions: on the one hand, the role of sending states as social protection providers (i.e. *provision role*) and on the other hand, their function of facilitating access to welfare for non-resident nationals (i.e. *facilitation role*).

We define sending states’ *provision role* as their ability to maintain a form of state-sponsored solidarity with the diaspora, either by allowing non-resident nationals to remain eligible from abroad for homeland-based social protection schemes or by creating special schemes specifically designed to address the welfare needs of this population. In volume 1 of this series (Lafleur and Vintila 2020a), we demonstrated that, within each one the five policy areas analysed here (i.e. unemployment, health, family, old-age, and economic hardship), there are important variations in the array of specific social benefits that Member States make available to different categories of (mobile and non-mobile) individuals. We further showed that the eligibility criteria for accessing such benefits often vary even within the same policy area. To enable the comparison between Member States’ policies towards their diaspora, we have therefore chosen in Table 1.2 to focus on one core benefit per policy area. Our analysis thus covers the following benefits: unemployment insurance benefits (depending on a qualifying period of contribution); contributory pensions (for individuals who reached the retirement age and/or sufficient years of contribution); family benefits (or “child benefits”, covering the costs of bringing up children); health benefits in kind (access to doctors, hospitalisation, treatment) and social assistance (means-tested benefits aiming to prevent poverty).

**Table 1.2:**
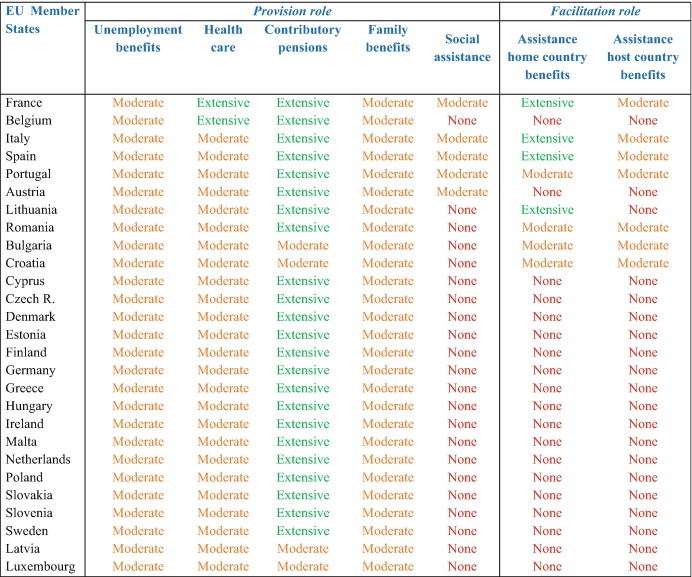
Substantive infrastructure: homeland policies responding to the social protection needs of diaspora populations

Source: Own elaboration based on MiTSoPro data. Regarding the *provision role*, the type of engagement for each benefit is categorised as follows: (a) unemployment benefits (extensive- worldwide exportability; moderate- exportability only for short periods when moving to EEA countries; none- no exportability); (b) health care (extensive- beyond EU legislation, additional scheme allowing non-residents to maintain homeland health insurance to cover medical treatment abroad or at home; moderate- medical treatment during short stays in the EU based on EHIC; none- no in-kind benefits for non-residents); (c) pensions (extensive- worldwide exportability; moderate- exportability in the EEA or based on bilateral social security agreements; none- no exportability); family benefits (extensive- worldwide exportability; moderate- exportability in the EEA or based on bilateral agreements; none- no exportability); social assistance (extensive- granted to nationals abroad, regardless of their host countries; moderate- conditional financial help in situation of economic hardship; none- no assistance for non-residents)

For each benefit, we consider that Member States that allow nationals residing abroad to access home country benefits regardless of where they live (in the EU, the European Economic Area (EEA) or in third countries) put forward an extensive form of engagement with the diaspora. At the opposite pole, countries that strictly restrict access to welfare entitlements to residence in their territory, thus automatically disqualifying non-residents from receiving such benefits, show no engagement with the social protection of their diaspora. Finally, Member States that do allow benefit exportability for non-resident nationals, but condition it to specific categories of individuals (such as those residing in particular countries) or to certain periods of time (only during short stays abroad), show only a moderate type of engagement. For this intermediary category, it is important to note that the EU legislation has pushed all Member States to adopt at least a moderate type of engagement with their diaspora. Indeed, the EU social security coordination framework made Member States more engaged with their nationals abroad in terms of recognition of the possibility to export certain benefits when leaving one’s country of nationality. This applies for almost all benefits analysed here, except for social assistance; although it is restricted only to nationals of EU Member States who move to other EU/EEA countries. As explained above, mobile EU citizens can continue to receive unemployment benefits for a short period when moving to another EU country with the purpose of finding a job. Similarly, they can receive medical treatment during short stays in another Member State based on the EHIC. The EU legislation also allows intra-EU migrants to receive contributory pensions from abroad, as well as family benefits in their EEA countries of residence, although the child resides in another EEA country. All these different situations in which EU nationals continue to enjoy social protection when moving abroad due to the EU legislation are categorized in Table 1.2 as moderate engagement, as they are always restricted in scope by covering only those moving to another EU/EEA country. Yet, some states have decided to take a step further in this regard by implementing diaspora-oriented social protection policies that go beyond this EU framework, thus putting forward an extensive engagement with their non-resident populations.

In addition to the *provision role*, the second important function that makes up sending states’ substantive infrastructure is the *facilitation role*, which refers to policies by which homeland authorities support citizens abroad in the administrative procedures to access home or host country welfare entitlements. It is therefore a policy-based commitment to facilitate access to social protection and an explicit recognition by homeland authorities that holding formal welfare rights in the home or host country is often not sufficient to access those rights in practice. Three important remarks need to be made regarding this definition of the *facilitation role*. First, unlike the previous sections of this chapter that looked exclusively at benefits delivered by the homeland, in this section we acknowledge that homeland authorities can play an active part also when it comes to helping nationals abroad to access welfare schemes granted by their residence countries. For this reason, Table 1.2 distinguishes between the *facilitation role* to access home country and host country benefits. Second, our analysis of the *facilitation role* focuses on the same benefits previously discussed for the *provision role*: unemployment benefits, health care, family benefits, social assistance, and pensions. Third, we consider as support the array of activities conducted by homeland authorities beyond mere information provision. As discussed in the country chapters, providing information on home/host countries’ welfare systems via websites and brochures, in person at consulates or even the facilitation of contacts of local NGOs and institutions active in the field of welfare is a very widespread practice EU Member States. In our view, active support however entails an intervention in citizens’ individual cases by providing personalized assistance and/or representation of interests in administrative dealings with welfare authorities. From this perspective, the delivery of life certificates by consulates or providing information on pensions on the consulates’ website, for example, cannot be considered as active support, but actual assistance to submit paperwork and ensure communication with pension authorities does qualify in this category.

To operationalise the level of support offered by homeland authorities to their diaspora, country experts examined the policies that define the missions of all institutions that compose each country’s descriptive infrastructure to determine if such support is part of their missions. Similarly to other indicators used to measure sending states’ substantive infrastructure, we identified three levels of engagement in the *facilitation role*. Sending states with policies that identify a specific responsibility of any institution to support nationals abroad in applying for any host/home country benefits are considered to offer extensive support. Sending states whose policies only mention a general principle of support in the area of welfare are considered to offer moderate support as this usually leaves significant room for discretion to actually implement such active assistance. Lastly, sending states whose policies do not even mention a principle of welfare-related support are considered to have a low level of engagement.

Keeping in mind these remarks, Table 1.2 compares EU Member States according to the benefits they provide for non-resident citizens (column on *Provision role*) and their engagement in facilitating diaspora’s access to welfare in home or host countries (column on *Facilitation role*). As observed, when it comes to the *provision role*, EU countries seem quite reluctant to extend welfare rights to their non-resident nationals. This goes in line with our previous findings (Vintila and Lafleur 2020) according to which, regardless of diaspora’s size or its economic and electoral leverage, EU Member States subscribe to a restrictive pattern that disqualifies non-residents from in-kind or cash benefits, as entitlement to most of these benefits remains conditional upon residence in the country. When benefit exportability is possible, this is generally driven by the EU legislation. As mentioned, thanks to the implementation of EU social security regulations, all Member States currently put forward at least a moderate level of engagement with their nationals abroad when it comes to the type of benefits granted to the diaspora. As shown in Table 1.2, with the exception of pensions which are generally exportable worldwide (with few exceptions of countries which allow pension exportability only to EEA countries, unless otherwise stipulated in bilateral agreements), very few Member States went beyond the EU legislation in granting social rights to non-resident populations. Interesting examples of pro-active diaspora engagement initiatives come from France and Belgium in the area of health care. As explained in the country chapters, these two Member States have set up special insurance schemes for their nationals moving to non-EU countries, allowing them to receive medical treatment either abroad or at home.

It is also interesting to note that, in the area of social assistance – which is not covered by the EU social security legislation-, most Member States have not implemented any financial assistance scheme for nationals abroad who are facing strong economic hardship beyond mechanisms of consular cash advances (sometimes non-reimbursable) usually designed to help citizens facing emergencies while temporarily abroad (e.g. tourists). Yet, France, Italy, Spain, Austria, and Portugal also offer some conditional type of economic support for citizens permanently abroad to help them deal with unpredictable medical issues and/or economic hardship. This type of support usually takes the form of (either recurrent or non-recurrent) non-reimbursable financial help, although it varies substantially in its scope, aims and claim procedure. For instance, recurrent non-contributory benefits can be delivered by consular authorities, as it happens with Austria’s Fund for the Support of Austrian Citizens Abroad or France’s fixed-term social allowance. In some cases, only specific groups qualify for such exceptional financial assistance. As illustrated in this volume, this is the case of Portuguese pensioners abroad who do not meet minimum subsistence levels and can apply for the “Social support for the deprived elderly of the Portuguese communities”.

As for the *facilitation role*, Table 1.2 demonstrates that France, Italy, and Spain represent the EU Member States that have assumed the most pro-active stance in facilitating the access of their nationals abroad to home or host country’s welfare benefits. The normative framework in these three countries clearly identifies an obligation for sending states’ authorities of different types to take an active role in the delivery of some homeland benefits. In the respective country chapters, this commitment is identified in the mission of France’s Consular Council, Italy’s Welfare Advice Agency and Spain’s Departments of Employment and Social Security at the consular level. On the other hand, Romania, Bulgaria, and Croatia put forward a more moderate engagement in this regard, as their consular policies only state a general commitment to support the diaspora to exercise social rights, without further details on the extent or content of such mission. Finally, Lithuanian authorities also provide assistance to nationals abroad to access welfare schemes from the home country, but not from the host. The other Member States do not provide any specific type of active support for facilitating non-residents’ access to welfare, apart from mere information on eligibility conditions for different types of social benefits.

Finally, although EU states’ policy responses towards their diaspora populations in the context of the COVID-19 pandemic fall outside of the scope of this volume, it is also important to note that many Member States have adopted an array of emergency measures for their citizens abroad in situation of need during this pandemic. Some of these measures were specifically intended to provide practical help to nationals abroad affected by the COVID-19 crisis (see examples of repatriation initiatives^17^), whereas in others, such measures focused on facilitating consular assistance and/or providing information regarding the social protection schemes of home and host countries.

## A Typology of EU Member States’ Social Protection Infrastructure for Citizens Abroad

At the outset of this introductory chapter, we postulated that existing research on diaspora policies does not take into consideration benefits and services deriving from the EU membership that protect EU citizens in situation of international mobility. When it comes to social protection, we showed in volume 1 of this series (Lafleur and Vintila 2020a) that, unlike other migrant groups, mobile EU citizens benefit from advanced access to their EU host countries’ welfare systems. With the concepts of descriptive and substantive infrastructure, this chapter therefore aimed to identify institutions and policies that— beyond the EU framework— provide an additional layer of protection for diaspora populations of EU Member States, whether they live inside or outside the EU.

**Fig. 1.5:**
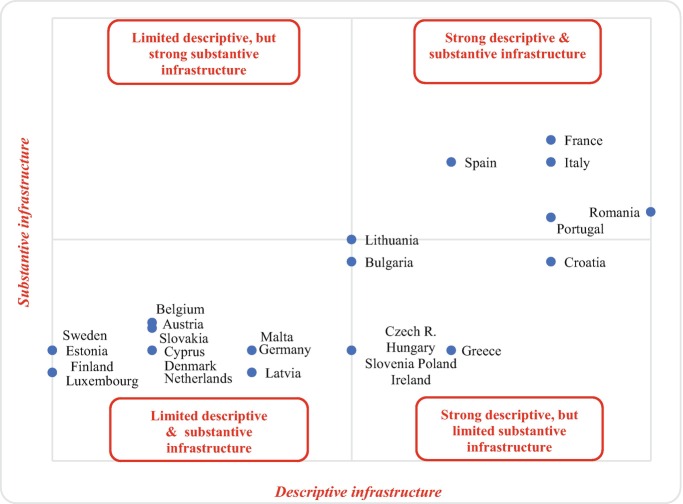
Typology of EU Member States by level of institutionalization and development of social protection policies for the diaspora Source: Own elaboration based on MiTSoPro data. The horizontal axis captures states’ descriptive infrastructure, calculated as an average of their network of consular, governmental and interest-representation institutions for the diaspora (Fig. 1.2). The vertical axis captures states’ substantive infrastructure, calculated as an average of their active engagement in the *provision role* and the *facilitation role* (Table 1.2)

Figure 1.5 summarizes our main findings regarding Member States’ performance in terms of descriptive and substantive infrastructures, thus aiming to generate a typology of sending states’ engagement with nationals abroad in the field of social protection.

The Figure allows us to draw several important conclusions. First, almost half of EU Member States return a limited descriptive and substantive diaspora infrastructure. This seems to indicate a strong disengagement with their non-resident populations, as these countries combine a limited institutional network for the diaspora with limited engagement in providing or facilitating their access to welfare. Yet, a closer look at the geographical distribution of their diaspora allows us to nuance this conclusion. To begin with, for six of those Member States (Austria, Cyprus, Belgium, Finland, Luxembourg, and Slovakia), most of their nationals abroad (up to more than 75% in some cases) concentrate in the EU. Hence, these countries may not perceive themselves as having global responsibilities towards their diaspora, especially since, by virtue of the EU citizenship status, most of their non-resident nationals are already protected in terms of access to welfare by EU regulations. Accordingly, these six Member States in particular are solely disengaged with a minority of their diaspora, namely those residing in non-EU countries. Of the remaining states in this first cluster, the country chapters demonstrate that some, which have a majority of non-resident nationals living outside the EU, are not necessarily less engaged. For instance, both Denmark and Sweden have Norway as a top non-EU destination for their diaspora and cooperate closely in the area of welfare with this country in the framework of the EEA and the Nordic agreements. Similarly, over one third of the Estonian and Latvian diaspora populations concentrate in the Russian Federation and are special minority groups with a particular status detailed in the respective country chapters. Lastly, Malta returns a limited descriptive and substantive infrastructure, although it has concluded advanced bilateral cooperation with the main non-EU destination countries of its diaspora. For instance, more than 40% of Maltese nationals abroad reside in Australia, but a bilateral agreement signed with this country ensures pensions payment abroad.^18^

Second, at the opposite end of the spectrum, a group of five EU Member States, including France, Italy, Spain, Portugal, and Romania, show a very strong engagement with their citizens abroad. All five countries combine extensive descriptive and substantive infrastructures for the social protection of non-resident nationals. In general, this position reflects a domestic political discourse about the importance of keeping ties with populations across the globe. Of these countries, Romania stands out as the EU Member State that, despite its relatively recent history of large-scale emigration, has put forward the most extensive network of descriptive infrastructure for its citizens abroad, which currently represent more than 15% of the country’ total population. However, unlike France, Italy or Spain, Romania returns a more moderate engagement in the facilitation of its diaspora’s access to homeland benefits, although this might be partially explained by the fact that most Romanians abroad (up to 85%) reside in other EU Member States where they already have access to social protection due to the EU citizenship status. Similarly, France also stands out in this cluster as the country with the strongest substantive infrastructure that allows its nationals abroad to keep accessing welfare benefits from France while residing outside Europe (see the discussion on the special insurance scheme for non-resident French in the corresponding country chapter).

A third cluster of countries combines a strong descriptive infrastructure with rather limited *provision* and *facilitation role* of sending states in ensuring non-residents’ social protection. The EU Member States included in this cluster seem to confirm the importance of the symbolic dimension of state-diaspora relations. In this case, a strong level of institutionalization of diaspora relations does not automatically lead to an extensive array of policies and services for citizens abroad. Country chapters on the Czech Republic, Greece, Lithuania, Poland, and Slovenia demonstrate clearly that the development of diaspora institutions has not been guided by welfare concerns, but rather by the desire to promote homeland identity abroad. In that strategy, social protection appears with a low priority, especially when compared to culture, education or citizenship. Ireland seems to be an outlier of this third cluster as despite its relatively high level of institutionalisation towards the diaspora, it has limited diaspora-oriented social protection policies. As discussed in the country chapter, this position can be explained by the fact that Ireland subcontracts its welfare missions to non-governmental actors in the main destination countries of its diaspora. Country chapters also illustrate the existence in other Member States of this kind of policy of funding migration organizations whom, in some cases, perform services of relevance to the diaspora in the area of welfare. Their activities, however, fall outside of the scope of our study on policies since, by definition, such organizations are not part of the sending states’ policy framework (i.e. not set in official norms) and cannot therefore be considered as a sending state response to the needs of the diaspora *stricto sensu*. Also, due to the fact that their funding is often limited in time and activities are oriented towards specific destination countries, it becomes difficult to draw any meaningful generalization from the observation of such activities.

Finally, this comparative overview also allows us to conclude that there is no EU Member State which has implemented extensive social protection policies for its diaspora without also having a well-developed institutional framework to engage with, consult or represent this population. This is visible in Fig. 1.5 by the absence of cases combining a strong substantive infrastructure with a limited descriptive infrastructure. In other words, states that aim to go beyond the EU framework in their diaspora protection policies tend to be those that have institutions that allow dialogue, contact and representation with this population. Lastly, the peculiar position of Lithuania at the centre of the graph deserves a word of explanation. Like most other Member States, Lithuania has a moderate substantive infrastructure with a dedicated institution at the sub-ministry level and a consultative body for diaspora affairs. Similarly, its engagement policies in the area of social protection are broadly limited to the EU framework. Yet, unlike in other Member States, the Lithuanian consular code identifies clear (but limited) responsibilities of its consulates in assisting citizens abroad to apply for some home country benefits.

The rest of the chapters included in this volume provide an in-depth analysis of EU Member States’ responsiveness to the social protection needs of their diaspora populations, by providing rich empirical examples of the repertoire of policies and programmes through which EU countries engage with their nationals residing abroad. After providing a short overview of the main characteristics of the diaspora of each EU Member State, country chapters critically examine the network of institutions that home countries authorities have designed for their nationals abroad. By highlighting their key engagement policies to address diaspora’s needs and by comparing the content of policies/services available to non-resident nationals, country chapters thus provide a detailed assessment of the centrality of social protection issues in the overall policy framework by which EU Member States dialogue with their populations abroad.

## Footnotes

1 Full text available here: http://legal.un.org/ilc/texts/instruments/english/conventions/9_2_1963.pdf. Accessed 16 March 2020.

2 Regulation (EC) No. 883/2004 of the European Parliament and of the Council of 29 April 2004 on the coordination of social security systems, https://eur-lex.europa.eu/legal-content/EN/ALL/?uri= CELEX:32004R0883. Accessed 16 March 2020. Regulation (EC) No. 987/2009 of the European Parliament and of the Council of 16 September 2009 laying down the procedure for implementing Regulation (EC) No. 883/2004 on the coordination of social security systems, https://eur-lex.europa.eu/legal-content/EN/ALL/?uri=CELEX:32009R0987. Accessed 16 March 2020.

3 For an analysis of the UK diaspora’s access to social protection, see Lafleur and Vintila (2020b) in this series.

4 Full text available here: https://eur-lex.europa.eu/legal-content/EN/TXT/?uri=CELEX%3A32015L0637. Accessed 16 March 2020.

5 For conditions of retiring abroad within the EU, see Regulation (EC) No. 883/2004, Regulation (EC) No. 987/2009 and https://europa.eu/youreurope/citizens/work/retire-abroad/index_en.htm. Accessed 16 March 2020.

6 See: International Convention on the Protection of the Rights of All Migrant Workers and Members of their Families, https://www.ohchr.org/EN/ProfessionalInterest/Pages/CMW.aspx. Accessed 16 March 2020.

7 See also COM (2012) 153 final- Communication from the Commission to the European Parliament, the Council, the European Economic and Social Committee and the Committee of the Regions- The External Dimension of EU Social Security Coordination, available here: https://eur-lex.europa.eu/legal-content/EN/ALL/?uri=CELEX: 52012DC0153. Accessed 16 March 2020.

8 Regulation (EC) No 883/2004 and Regulation (EC) No 987/2009. See also: https://europa.eu/youreurope/citizens/work/unemployment-and-benefits/unemployment/index_en.htm. Accessed 16 March 2020.

9 For coordination of family benefits, see Regulation (EC) No 883/2004, Regulation (EC) No 987/2009 and https://europa.eu/youreurope/citizens/work/unemployment-and-benefits/family-benefits/index_en.htm. Accessed 16 March 2020.

10 See: https://ec.europa.eu/social/main.jsp?catId=559&langId=en. Accessed 16 March 2020.

11 In this body of literature, descriptive political representation traditionally focuses on minority empowerment by postulating that the composition of representative institutions should “mirror” the composition of the population it aims to represent. On the other hand, substantive representation captures the extent to which elected office holders are responsive to the specific interests, needs, demands and preferences of the group they represent.

12 http://labos.ulg.ac.be/socialprotection/. Accessed 16 March 2020. The surveys were conducted between April 2018–January 2019 and several rounds of consistency check were centrally conducted by the MiTSoPro team. Given the period in which the surveys were conducted, the country chapters included in this volume focus mainly on the policies in place at the beginning of 2019.

13 In some countries (Spain, Italy, Germany, Portugal or Belgium), sub-national level authorities also develop policies towards the diaspora. While this research focuses on national-level policies, examples of such sub-national policies are provided for illustrative purposes in the respective country chapters.

14 Honorary consulates frequently perform a symbolic role in representing a state’s interests abroad and are often run by non-professional diplomats. In certain cases, they also offer limited administrative services to citizens abroad. It should also be noted that, in certain countries, what we refer here with the generic term of honorary consulate is called differently (e.g. royal consulates in Denmark).

15 This criterion allow us to exclude Ministry of Foreign Affairs’ consular affairs departments that are present across all EU Member States.

16 Austria, Bulgaria, Croatia, the Czech Republic, France, Greece, Hungary, Ireland, Italy, Lithuania, Malta, Poland, Portugal, Romania, Slovenia, Spain.

17 See also: https://eeas.europa.eu/headquarters/headquarters-homepage/76203/good-stories-consular-support-eu-citizens-stranded-abroad_en; https://www.europarl.europa.eu/RegData/etudes/BRIE/2020/649359/EPRS_BRI%282020%29649359_EN.pdf (accessed 19 May 2020).

18 https://www.dss.gov.au/about-the-department/international/international-social-security-agreements/current-international-social-security-agreements/australia-and-malta-frequently-asked-questions. Accessed 16 March 2020.
